# Statin drugs mitigate cellular inflammatory response after ST elevation myocardial infarction, but do not affect in-hospital mortality

**DOI:** 10.15171/jcvtr.2016.06

**Published:** 2016-03-15

**Authors:** Leili Pourafkari, Ognjen Visnjevac, Samad Ghaffari, Nader D. Nader

**Affiliations:** ^1^Cardovascular Research Center, Tabriz University of Medical Sciences, Tabriz, Iran; ^2^State University of New York at Buffalo, Buffalo, NY, USA

**Keywords:** Myocardial Infarction, Statins, Hyperlipidemia, Heart Failure

## Abstract

***Introduction:*** The objective was to examine the role of statins in modulating post-STEMI inflammation and related mortality.

***Methods:*** A total of 404 patients with STEMI were reviewed. Demographics, comorbidities, laboratory values, and outcomes were collected. The patients were grouped as STATIN and NOSTAT based on the use of statin drugs at the time of admission. Ninety-seven patients were receiving statin drugs.

***Results:*** The patients in the STATIN group were more likely to be hypertensive (53.6%), diabetic (37.1%) and to have previous coronary revascularization (9.3%). Following propensity matching of 89 patients in STATIN group to an equal number of patients in NOSTAT controls had lower neutrophil count 7.8 (6.8-8.4) compared to those in the NOSTAT group 9.1 (7.9-10.1). Although there was no difference in-hospital mortality between the two groups, the incidence of pump failure was lower in the STATIN group (5.6% vs. 15.7%; *P* < 0.01).

***Conclusion:*** Statin treatment prior to STEMI mitigates the cellular inflammatory response after the myocardial infarction, as evidenced by lower leukocyte and neutrophil cell counts in the STATIN group.

## Introduction

Acute ST elevation myocardial infarction (STEMI) is associated with a significant local inflammatory response is initiated in the myocardium and propagates systemically into the blood stream. Proximal inflammatory cytokines such as tumor necrosis factor alpha (TNFa) and variety of chemokines are scant in healthy hearts, however their levels spike up during myocardial infarct.^1-3^

Elevation of acute phase reactants such as C-reactive protein and increased peripheral white blood cell count, especially neutrophils, are common manifests of systemic inflammation during an acute coronary syndrome.^4,5^ Various investigators have reported associations between increased neutrophil count in peripheral blood and short-term post-STEMI adverse outcomes, including mortality.^6-8^ It has been imperative for both clinicians and reseachers in the field of cardiovascular medicine to successfully mitigate the exuberent inflammatory responses during an acute coronary event with the aim of limiting the extent of the myocardial injury and thereby improving the outcome following STEMI.

The use of statins (3-hydroxy-3-methylglutaryl coenzyme A reductase inhibitors) has been associated with atheroprotective and anti-inflammatory effects, independent of their lipid lowering effects.^9-11^ Their pharmacological action has been attributed to improvements in endothelial function, reduction in smooth muscle cell proliferation and migration and reduction in the number of smooth muscle cells along with a reduction in the collagen content in plaques.^12,13^ In the Heart Protection Study, the largest randomized study on the effects of statins on major morbidity and mortality, the authors reported that all-cause mortality was reduced in patients who received statins, primarily due to the reduction in death from vascular causes.^14^ There have also been reports on the effects of statin drugs on reducing postoperative morbidity and mortality following various cardiovascular procedures.^15-17^

The goal of this study was to determine if the use of statin drugs was associated with a decrease in inflammatory response, adverse events, and mortality in patients admitted to the coronary care unit with a newly diagnosed STEMI. We hypothesized that the patients who were on statins at the time of the event had more limited inflammatory response and better survival following a STEMI.

## Patients and Methods

After obtaining informed consent, a total of 404 patients who were admitted to the Coronary Care Unit with acute STEMI were enrolled from March 2010 to March 2012. Patients were assigned to the statin (STATIN) group, or the no statin (NOSTAT) group depending on whether they were receiving statin therapy at the time of the STEMI, or not. Patients who were on statins at the time of STEMI were already started on these drugs by their primary providers, but the exact duration of statin drug use was not available for this study. Patients with active inflammation or chronic inflammatory diseases, past history of surgery within 3 months prior to STEMI, and cancer were excluded from the study. STEMI was determined using the definition and criteria provided by American College of Cardiology and European Society of Cardiology; in short: STEMI was defined as an increase in cardiac troponin-I (cTNI) along with new ST-segment elevation measured from J point at least 0.2 mV in two adjacent V1-V3 leads or at least 0.1 mV in other leads within 24 hours after the onset of chest pain.^18^

The information regarding demographic features, past medical history, patient outcomes and the occurrence of major complications or mortality were collected from the medical records for all subjects. Complete blood cell count was performed in all patients within 24 hours of onset of symptoms. Blood samples were evaluated for total leukocyte, neutrophil, lymphocyte and monocyte count using SE-2100 Scattergram (Sysmex, Mundelein, IL). The neutrophil lymphocyte ratio was calculated for each patient. The following laboratory values were collected for all patients: hematocrit, creatinine, creatine kinase (CK) with MB fraction (CKMB) and cTNI. The peak values were documented and reported for all laboratory variables.

Hypertension was defined as blood pressure of ≥140/90 mm Hg recorded at least two times or current antihypertensive therapy. Diabetes was defined as fasting plasma glucose of >126 mg/dL for at least two measurements or current glucose lowering treatment as defined by the World Health Organization (WHO).^19^ Renal insufficiency was defined as having a serum creatinine higher than 1.5 mg/dL or greater, or a patient on dialysis. Hyperlipidemia was defined as fasting cholesterol level >200 mg/dL, a low-density lipoprotein level >130 mg/dL, or triglycerides >200 mg/dL before starting on lipid-lowering therapy.

### Statistical analyses and data management

Data analysis was performed using SPSS 18.0 software (SPSS Inc, Chicago, IL) and NCSS (Kaysville, Utah). Primary endpoints were peak leukocyte and neutrophil counts, and in-hospital mortality. Secondary endpoints included pump failure (defined as cardiogenic shock and/or pulmonary edema), ICU length of stay and major tachyarrhythmias (defined as atrial fibrillation and ventricular tachycardia or fibrillation).

Demographic comparisons were made using chi-square, or two-tailed Fisher’s exact test for categorical variables, and by Wilcoxon Rank-Sum test for continuous variables without normality distribution. Univariate analysis was performed using log-rank test for identifying factors predicting in-hospital mortality. All factors that show a trend of significance as identified by *P* values <0.1 in univariate analyses were included in multivariate analyses. Propensity scores were calculated using hypertension, diabetes, unstable angina and previous coronary revascularization. Since the majority of hyperlipidemia patients were in the STATIN group, this variable was not used for matching. Doing so would have eliminated majority of the patients who were receiving statins. Data were then matched (1:1) using the calculated propensity scores for the STATIN cohort and NOSTAT controls. Odds ratios were reported for 2×2 tests and 95% confidence interval for event endpoints analyses. Null hypotheses were rejected if *P* values were less than 0.05.

## Results

Of the 404 patients enrolled in this study, 97 were taking statins at the time of STEMI. Patients in the STATIN group had significantly more hyperlipidemia, hypertension, diabetes mellitus, and prior revascularizations along with a higher blood glucose concentration on admission. After propensity matching, there were no statistical differences in demographics, comorbidities, or laboratory values between the two groups, except for hyperlipidemia, which remained statistically higher in the STATIN group as this variable was not included in the propensity matching calculation (Table 1).

**Table 1:** Patient demographics, comorbidities, and laboratory data

**Variable**	**Before Propensity Match**			**After Propensity Match**		
	**STATN ** **Sn=97**	**NOSTAT ** **Sn=307**	***P *** ** value**	**STATN** **n=89**	**NOSTAT** **n=89**	***P *** ** value**
Age*	61.5[56-65]	57.0[55-60]	0.32	61(56-65)	60[56-65]	0.50
Female**	23(23.7)	53(17.3)	0.16	22(24.7)	23(25.8)	0.86
Family history	24(24.7)	63(20.5)	0.38	22(24.7)	13(14.6)	0.09
Hyperlipidemia	82(84.5)	32(10.4)	<0.01	81(91)	13(14.6)	<0.01
Hypertension	52(53.6)	105(34.2)	<0.01	47(52.8)	46(51.7)	0.88
Diabetes mellitus	36(37.1)	65(21.2)	<0.01	34(38.2)	35(39.3)	0.88
Active smoker	36(37.1)	144(46.9)	0.09	35(39.3)	32(36.0)	0.64
Stroke	4(4.1)	17(5.5)	0.59	4(4.5)	9(10.1)	0.15
Unstable angina	3(3.1)	26(8.5)	0.07	3(3.4)	3(3.4)	1.00
Myocardial infarction	11(11.3)	21(6.8)	0.15	8(9.0)	7(7.9)	0.79
Prior revascularization	9 (9.3)	2 (0.7)	<0.01	1(1.1)	1(1.1)	1.00
LVEF*	40[35-40]	40[40-40]	0.82	40[35-40]	40[30-40]	0.29
Length of hospital stay (days)	7[6-7]	6[5-6]	0.06***	6[6-7]	6[6-7]	0.89
Hematocrit %	43.6[42.3-44.7]	43.0[42.3-43.8]	0.44	43.7 [42.3-44.7]	42.1[41.0-43.5]	0.09***
Creatinine mg/dL	0.93[0.85-1]	0.93[0.9-0.97]	0.75	0.93[0.85-1]	0.97[0.9-1]	0.37
Blood glucose (mg/dL)	157[136-180]	142[134-146]	0.05	156[135-174]	155[136-175]	0.71

* Median [range]; Wilcoxon Rank-Sum 2-tailed test used to calculate *P* value. ** n (%); Chi-squared used to calculate *P* value. *** Although 2-tailed test showed non-significance, a 1-tailed test indicated significance

Before propensity matching, sequelae following STEMI were similar between the two groups except for peak cTNI, which was higher in the NOSTAT group (4.6 ng/ml, 95% CI: 3.2-6.4) than in the STATIN group (2.3 ng/ml, 95% CI: 0.9-4.5, *P*=0.043). CK and CKMB both displayed a strong trend toward significance, having non-significant p-values with a 2-tailed analyses and statistical significance with the 1-tailed analyses (Table 2). After propensity matching, these markers of cardiac ischemia were no longer found to be statistically different between groups, but there was a lower incidence of pump failure (OR=0.39, 95% CI: 0.11-0.93, *P*=0.036) (Table 3). Overall in hospital mortality was low (15 patients, 3.71% of total sample) and it was not significantly different between the STATIN (OR=0.59, 95% CI: 0.14-2.53, *P*=0.474) and NOSTAT groups.

**Table 2:** Post-STEMI sequelae (not propensity matched)

**Clinical/laboratory outcome**	**STATN ** **n=97**	**NOSTAT ** **n=307**	***P *** **value**
Ventricular Dysrhythmias within 24 hours**	4(4.1)	25(8.1)	0.181
Ventricular Dysrhythmias after 24 hours**	2(2.1)	6(2.0)	0.947
New right bundle branch block**	8(8.2)	32(10.4)	0.532
New left bundle branch block**	5(5.2)	9(2.9)	0.297
Atrial fibrillation**	5(5.2)	15(4.9)	0.915
Paroxysmal supraventricular tachycardia**	0(0)	6(2.0)	0.165
1st or 2nd degree AV block**	5(5.2)	19(6.2)	0.934
Complete AV block**	2(2.1)	4(1.3)	0.590
Ventricular septal defect**	1(1.0)	1(0.3)	0.388
Mitral regurgitation**	7(7.2)	20(6.5)	0.809
Pulmonary edema**	3(3.1)	18(5.9)	0.284
Pump failure**	6(6.2)	29(9.4)	0.320
Repeat in-hospital myocardial infarction**	1(1.0)	3(1.0)	0.963
In-hospital mortality**	4(4.1)	11(3.6)	0.806
Creatine kinase (units)*	524[282-1038]	922[692-1199]	0.070***
Creatine kinase MB isoenzyme (units)*	60 [47-89]	91 [72-112]	0.080***
Cardiac troponin I (nanogram/mL)*	2.3[0.9-4.5]	4.6[3.2-6.4]	0.043

* Median [range]; 2-tailed Wicoxon Rank-Sum test used to calculate P value. ** n (%); Chi-squared used to calculate P value. *** Although 2-tailed test showed non-significance, a 1-tailed test indicated significance

**Table 3:** Post-STEMI sequelae (propensity matched)

**Clinical/Laboratory Outcome**	**STATN ** **N=89**	**NOSTAT ** **N=89**	**P-Value**
Ventricular dysrhythmias within 24 hours**	3(3.4)	5(5.6)	0.469
Ventricular dysrhythmias after 24 hours**	2(2.2)	1(1.1)	0.561
New right bundle branch block**	7(7.9)	12(13.5)	0.225
New left bundle branch block**	4(4.5)	3(3.4)	0.700
Atrial fibrillation**	5(5.6)	6(6.7)	0.756
Paroxysmal supraventricular tachycardia**	0(0)	1(1.1)	0.316
1st or 2nd degree AV block**	5(5.6)	5(5.6)	1.000
Complete AV block**	2(2.2)	2(2.2)	1.000
Ventricular septal defect**	1(1.1)	0(0)	0.316
Mitral regurgitation**	7(7.9)	4(4.5)	0.350
Pulmonary edema**	3(3.4)	10(11.2)	0.044
Pump failure**	5(5.6)	14(15.7)	0.023
Repeat in-hospital myocardial infarction**	0(0)	1(1.1)	0.316
In-hospital mortality**	3(3.4)	5(5.6)	0.469
Creatine kinase (units)*	601[353-1126]	889[504-1204]	0.386
Creatine kinase MB isoenzyme (units)*	61 [49-94]	81 [63-119]	0.780
Cardiac troponin I (nanogram/mL)*	2.5 [1.2-6.2]	6.2 [1.5-10.4]	0.145

* Median [range]; 2-tailed Wicoxon Rank-Sum test used to calculate *P* value. ** n(%); Chi-squared used to calculate *P* value. *** Although 2-tailed test showed non-significance, a 1-tailed test indicated significance

The inflammatory response following STEMI was statistically different before and after propensity matching, with a post-matched increase in leukocyte (*P*=0.001) and neutrophil (*P*=0.002) count in the NOSTAT group (Table 4). There were no differences in monocyte or lymphocyte counts and the neutrophil-lymphocyte ratio was not statistically different between groups after propensity matching (Table 4). Neutrophil count was not associated with an improvement in survival (OR=0.95, 95% CI: 0.30-3.00, *P*=0.925)

**Table 4:** Leukocytic inflammatory response following STEMI

**Variable**	**Before Propensity Match**			**After Propensity Match**		
	**STATN ** **N=97**	**NOSTAT ** **N=307**	***P *** **value**	**STATN ** **N=89**	**NOSTAT ** **N=89**	***P *** **value**
Total leukocyte count	9.9[9.2-10.7]	11.0[10.6-11.6]	0.001	9.9[9.0-10.7]	11.6[10.6-12.7]	0.001
Neutrophils	7.3[6.7-8.4]	8.6[8.1-9.1]	0.001	7.8 [6.8-8.4]	9.1[7.9-10.1]	0.002
Lymphocytes	1.4[1.3-1.6]	1.4[1.3-1.4]	0.364	1.4[1.2-1.6]	1.4[1.2-1.6]	0.875
Monocytes	0.61[0.53-0.67]	0.62[0.58-0.65]	0.402	0.6[0.52-0.67]	0.61[0.57-0.70]	0.314
Neutrophil/lymphocyte	5.0[4.0-6.1]	5.9[5.2-6.6]	0.035	5.2[4.5-6.2]	6.2[4.8-7.8]	0.181

Values presented as Median [range]; 2-tailed Wilcoxon Rank-Sum test used to calculate *P* value.

## Discussion

Statin drugs were found to mitigate the cellular inflammatory response after ST elevation myocardial infarction, as evidenced by statistically lower leukocyte and neutrophil cell counts in the STATIN group, without evidence of differences in monocyte or lymphocyte cell counts. This is an important finding as it may provide the pathophysiologic evidence that anti-inflammatory effects of statins and mortality benefit are the direct result of mitigated neutrophil activation during acute coronary syndromes. Although the effect of statins on in-hospital mortality could not effectively be examined in this study because of its low occurrence rate (3.71% of the overall sample) and relatively short follow-up period, other studies have provided strong evidence that statins carry a mortality benefit and that peripheral neutrophilia is associated with worse cardiovascular outcomes, with this study providing evidence of a connection between the two (statins and neutrophilia).^6-8,14^

Associated neutrophilia following acute myocardial infarction may indeed be related to decreased margination of neutrophils.^20^ This phenomenon has been ascribed in these patients due to increased circulating levels of catecholamines. The effects of statin drugs on endothelial cell function and vascular smooth muscles may restore the margination of the neutrophils and contribute to the lower count of these inflammatory cells in the peripheral blood.^21^ Although the contribution of statins to the endothelium-neutrophil interaction during STEMI is beyond the scope of this study, this mechanism of action may have some theoretical value. Cholesterol-independent or “pleiotropic” effects of statins are thought to protect against myocardial injury and may occur via a number of mechanisms including their effect on endothelial function.^22^

A recent systematic review of the routine use of high-flow oxygen in the treatment of uncomplicated myocardial infarction found that its routine use might result in a greater infarct size and increase the risk of mortality.^23^ These findings are supported by direct physiologic evidence of the phenomenon of hyperoxia-mediated coronary vasoconstriction, but hyperoxic-cardiac injury may not be limited to this mechanism.^24^ Hyperoxia also affects the redox cellular state of ischemic myocardium, fuels the abundance of reactive oxygen species generated by the neutrophilia of acute coronary syndromes, and has been implicated in exacerbating oxygen radical-mediated reperfusion injury following coronary revascularization.^25^ The concurrent effect of statin use for mitigating elements of hyperoxic-injury in the context of neutrophilia and oxygen radical formation was not assessed in our study, nor any other to date.

This study also found a higher incidence of pump failure in the NOSTAT group, but the etiology of this remains unclear. Although a greater degree of myocardial injury, as evidenced by higher mean blood concentrations of cardiac markers of ischemia in the NOSTAT group (Table 3), may infer a logical explanation, the wide range of cardiac marker values in this population sample yielded non-statistically significant differences between the two study groups. There were no statistically significant differences in major tachyarrhythmias, new-onset conduction delays, new-onset mitral regurgitation, or recurrent in-hospital myocardial infarction between the two groups. The extent of mitral regurgitation was not documented, however, and may have contributed to the higher incidence of pump failure and pulmonary edema in the NOSTAT group.

There were several limitations to this study. First, during propensity matching process, it was evident the majority of patients diagnosed with hyperlipidemia were in the statin group and could not be effectively matched without excessively compromising the post-propensity matched sample size. It is also possible that there were patients without this hyperlipidemia diagnosis in each group that were, in fact, hyperlipidemic but had not yet been diagnosed as such.

Second, this study’s primary endpoint of in-hospital mortality yielded low numbers and limited statistical analysis. Others have described long-term mortality to be significantly higher than was found in this study, but this study did not follow patients or assess their outcomes following hospital discharge.^26^ Furthermore, the post-STEMI day of mortality was not recorded in this study, nor was there any assessment of all-cause mortality following hospital discharge.

This study’s major finding was that statins mitigate the cellular inflammatory response following STEMI which directly influences and focuses future research. The subcellular and intercellular pathways by which statins exert this effect need to be elucidated. Neutrophil mediated cellular injury is a function of blunt mechanisms (oxygen radical production, lytic enzyme utilization, and phagocytosis) combined with directed extravasation and targeting, identifying several potential areas of immunologic modifiable risk factors in the post-STEMI period.^27^ Statins’ effects on neutrophil-mediated reperfusion injury are also yet to be elucidated.

## Ethical issues

This study was approved by the Institutional Review Board of Tabriz University of Medical Sciences for its scientific and ethical merit.

## Competing interests

None.
